# Structure Determination of Er Doped Ti-Al-Nb Alloy by Neutron Diffraction Analysis

**DOI:** 10.3390/ma12142243

**Published:** 2019-07-12

**Authors:** Yubin Ke, Juzhou Tao, Huiping Duan

**Affiliations:** 1 School of Materials Science and Engineering, Beihang University, Beijing 100191, China; 2 Dongguan Neutron Science Center, Institute of High Energy Physics, Chinese Academy of Sciences, Dongguan 523808, China

**Keywords:** titanium aluminide alloy, rare-earth alloying, mechanical properties, neutron diffraction, stress, strain

## Abstract

Neutron diffraction experiments with both conventional powder diffraction setup and under in-situ compressive loading were conducted to investigate the structural origins of large strength and toughness enhancements in Ti-16Al-27Nb alloy after Er addition. The primary phase is determined to be the ordered B_2_ structure form, in agreement with the previous electron microscopy study. Lattice strains of {210} and {100} planes were measured as a function of applied stress, and elastic anisotropy was found for both, and strong plastic nonlinearity was discovered for (210) reflection. The grain refinement during plastic deformation was proposed by both the 2D diffraction intensity distribution and SEM observations, while stress-induced martensitic phase transition was not observed in this study. It is believed that the activation of different slip systems and grain refinement might be the structural origin of the novel mechanical properties of this alloy.

## 1. Introduction

Due to its high yield stress at elevated temperature, excellent ductility and strong oxidation resistance, Ti-Al-Nb ternary alloys have been actively developed as a new high temperature structural material for aerospace and power industries. More recently, a good combination of strength and ductility was realized in an alloy with composition Ti-(22-26) at.%Al-(15-30) at.%Nb [1,2]. However, the low toughness, ductility and poor machinability of this alloy at room temperature exist as major material disadvantages and limit its application [3]. In previous research work, we added rare earth (Erbium) into a Ti-16Al-27Nb alloy and found large increases in room temperature fracture stress, and toughness increased as much as three times [4]. One possible mechanism for such remarkable improvement in these mechanical properties is the stress-induced martensitic transformation (SIMT) under tensile loading, previously reported for Ti-15Al-12Nb, Ti-15Al-8Nb and Ti-18Al-8Nb [5]. While the measured macroscopic compressive stress-strain curve of the Ti-16Al-27Nb alloy [4] has a very similar shape to those of the other Ti-Al-Nb alloys and is consistent with a SIMT mechanism, the model lacks direct experiment evidence as no β to α’’(martensite) phase was observed for Er added Ti-16Al-27Nb in an electron microscopy study. It is possible that poor electron scattering contrast between the martensitic and other phases, and/or the complicated TEM specimen preparation process, make the phase difficult to identify. Here, we report neutron diffraction experiment results of the Er added alloy, as both an in situ microstructure characterization and an attempt to detect the possible stress-induced martensitic phase transition.

Previous structure characterization of as-cast Ti-Al-Nb sample by TEM establishes three phases in the alloy: the primary β/B_2_ phase based on disordered bcc Ti structure or ordered B_2_ structure (CsCl type) [6]; a secondary close-packed orthorhombic O phase covering a range of compositions around Ti_2_AlNb stoichiometry which is a transformation of the high temperature β/B_2_ phase involving no long range diffusion [7]; and a small amount of bcc rare earth precipitate phase (AlTiNb)_2_Er_3_ [4]. If the as-cast alloys were annealed, hexagonal close-packed phase α_2_ with a range of compositions around Ti_3_Al also appears through phase transformation paths closely related to that of B_2_ to O transformation [8,9]. The ideal B_2_ structure consists of two cubic sub-lattices displaced by the bcc lattice vector (1/2, 1/2, 1/2). Structural relations between different phases in the ternary Ti-Al-Nb alloy system can be viewed as variations of B_2_ lattice plane distortion and shuffle, changes in interplanar distances, and ordering/disordering of Ti, Nb and Al atoms among the two lattice sites [10,11]. In this paper, in situ observation of the crystal structure and microstructure of Er doped Ti-16Al-27Nb Alloy under stress loading were conducted. The experimental results can give an insight to the structural origin of the novel mechanical properties of this alloy.

## 2. Materials and Methods

Starting with high purity (over 99.9%) Ti, Al, Nb and Er metals, Ti-Al-Nb ingots with and without 0.6 wt.% Er addition were fabricated by arc melting in a copper crucible under an argon atmosphere. The ingots were re-melted six times to ensure chemical homogeneity. Microstructure of the materials was characterized by a Rigaku X-ray powder diffractometer (XRD) with Cu Kα radiation and scanning electron microscopy (SEM). Samples for XRD, SEM, neutron powder diffraction (NPD) investigations and mechanical property tests were all cut from the ingots and mechanically ground with SiC abrasive papers up to 2000 grid. The samples for SEM measurement were end-polished with Fe_2_O_3_ water solution. The samples for NPD experiments were cut in a rod shape with 6 mm, 7 mm and 8 mm diameters. Each sample rod has a length of approximately 1.5 times of the respective diameter. The metallographic samples were etched with a mixture solution with HF: HNO_3_: H_2_O_2_:H_2_O = 1:2:5:25 at room temperature. 

Neutron powder diffraction measurements were conducted at the OPAL reactor source of the Bragg Institute, Australian Nuclear Science and Technology Organization (ANSTO, Sydney, Australia). High intensity neutron powder diffractometer WOMBAT was used for quick scans of NPD pattern at λ = 2.41 Å incident beam wavelength covering 2θ scattering angle between 15° and 136° at 0.125° step size. High resolution neutron powder diffractometer ECHIDNA was used to collect data for structure refinement at λ = 1.6215 Å with 2θ between 4° and 164° at 0.05° increment. Samples were rotated about the Ω axis perpendicular to the diffraction plane for the ECHIDNA measurements. Neutron powder diffraction experiments under in situ stress loading were conducted with the residual stress diffractometer KOWARI. 210 and 100 reflections were chosen because of both having relatively strong peak intensity and large enough scattering angle to accommodate for instrument setup. The KOWARI load frame was set up for uniaxial compressive stress control up to 1000 MPa followed by strain/position control of 0.1 mm increment up to 3 mm sample deformation. The diffractometer KOWARI setup is in Laue geometry to ensure that measured scattering vector is parallel to the axial loading direction.

Lattice strain is defined as

(1) $$\frac{d_{\mathrm{hkl}}-d_{\mathrm{hkl}}^{0}}{d_{\mathrm{hkl}}^{0}}$$

where $d_{\mathrm{hkl}}$ and $d_{\mathrm{hkl}}^{0}$ are measured lattice spacing for deformed and undeformed sample. Structure refinement with neutron diffraction data uses the GSAS program suite [12].

## 3. Results and Discussion

**Figure 1:**
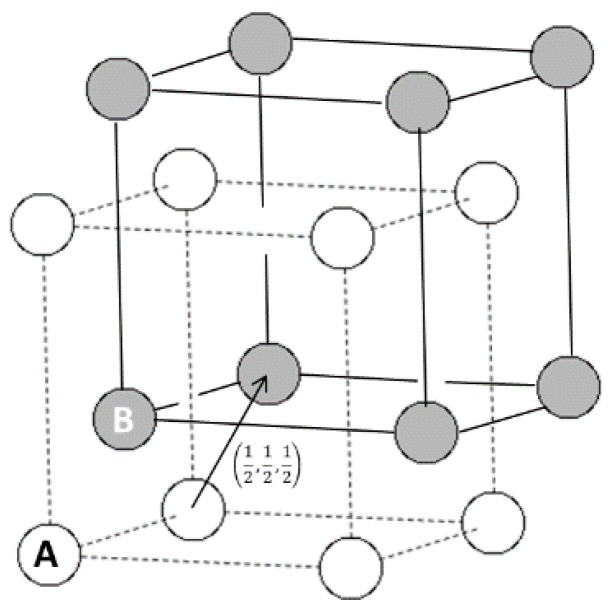
The structure of the ordered B_2_ phase. Open (white) and filled (gray) spheres show the A and B lattice sites. Two sub-lattices are displaced by the bcc translation vector.

Bragg scattering peaks observed in XRD and NPD experiments of the same Ti-16Al-27Nb-0.6Er as-cast alloy can be consistently indexed by a cubic cell. Only h + k + l = even peaks are clearly observed in XRD, whereas both h + k + l = odd (weak) and h + k + l = even (strong) peaks are present in NPD. In the ideal B_2_ structure based on titanium aluminide intermetallics, as shown in Figure 1, two sub-lattices can be denoted as A and B. Sub-lattice A is exclusively occupied by Ti and sub-lattice B is randomly filled by Ti and Al. Since Nb has about the same atomic radii as Ti and is a known β/B_2_ phase stabilizer [13], we assume Nb randomly substitutes for Ti sites in the Ti-16Al-27Nb alloy. 

The structure factor of such a B_2_ unit cell is then:

(2) $$F_{\mathrm{hkl}}=f_{A}+f_{B}e^{\pi i\left(h+k+l\right)}$$

**Figure 2:**
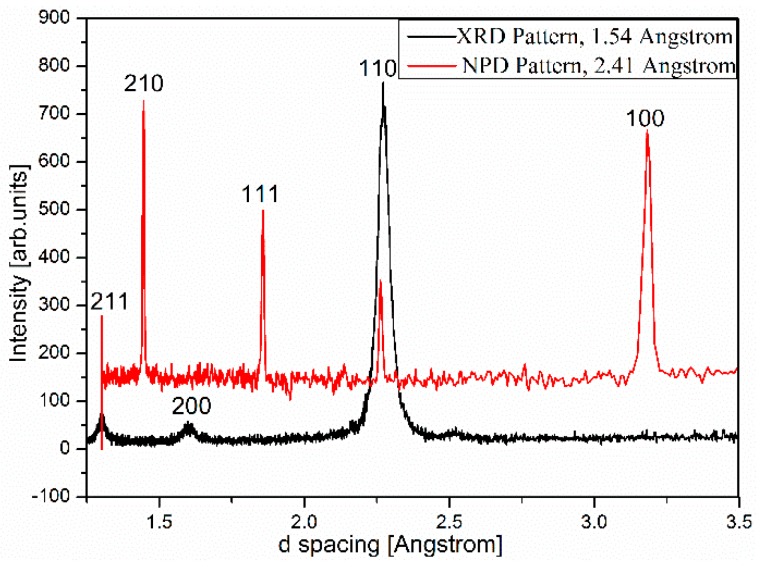
XRD and neutron powder diffraction (NPD) (measured with WOMBAT diffractometer) patterns of the Ti-16Al-27Nb-0.6Er alloy.

Site scattering factors $f_{A}$ and $f_{B}$ are statistical averages of atomic scattering amplitudes based on distribution of atoms in lattice sites A and B. For XRD, both site scattering factors are positive, therefore we expect strong diffraction peaks with h + k + l = even and weak peaks when h + k + l = odd. Given that Ti is the predominant element in Ti-16Al-27Nb with negative neutron scattering length, the situation is reversed for NPD resulting in two site scattering factors of opposite signs. Consequently h + k + l = odd reflections are strong peaks and h + k + l = even peaks are weak. The fact that we did not observe weak h + k + l = odd peaks in XRD pattern is likely due to high background of XRD data. The measured XRD and NPD patterns with peak reflection indexes are shown in Figure 2.

Ti-16Al-27Nb-0.6Er X-ray data are collected on a powder diffractometer with a Cu Kα source, neutron data are from the WOMBAT diffractometer (Sydney, Australia.) with the incident beam wavelength at 2.41 Å.

Rietveld refinement using the high resolution NPD data was performed based on the B_2_ structure model described above. First level refinement establishes three background parameters, one scale parameter, one diffractometer zero constant and one cubic cell parameter. Subsequent refinements fix site occupancies and four peak profile parameters related to Gaussian and Lorentzian broadening of peaks due to grain size. The results of the structure refinement are summarized in Table 1 and confirm our initial guess: A (0,0,0) sites are randomly occupied by Ti or Nb almost in equal chance; some B (1/2, 1/2, 1/2) sites are taken by Al with the rest filled by Ti. The reason for a very small fraction of Nb at B site (2%) is unclear. 

**Figure 3:**
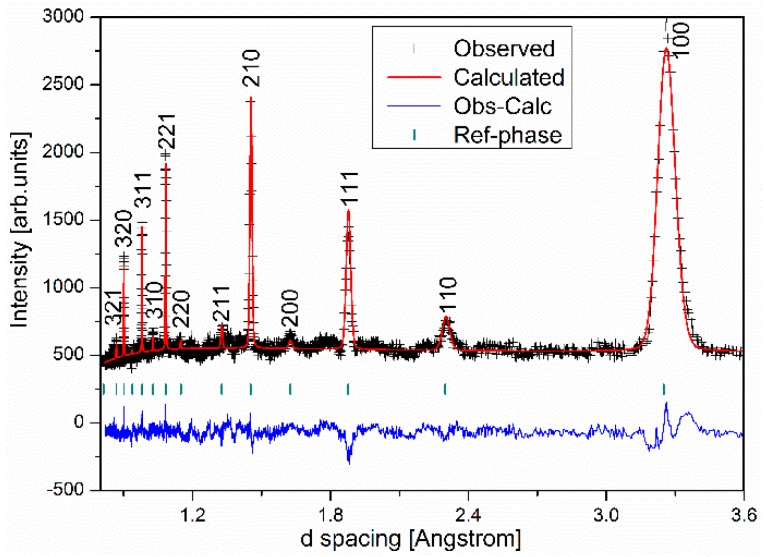
Structure refinement with neutron diffraction data (measured with ECHIDNA diffractometer).

The agreement between the observed and calculated intensities is shown in Figure 3.

**Figure 4:**
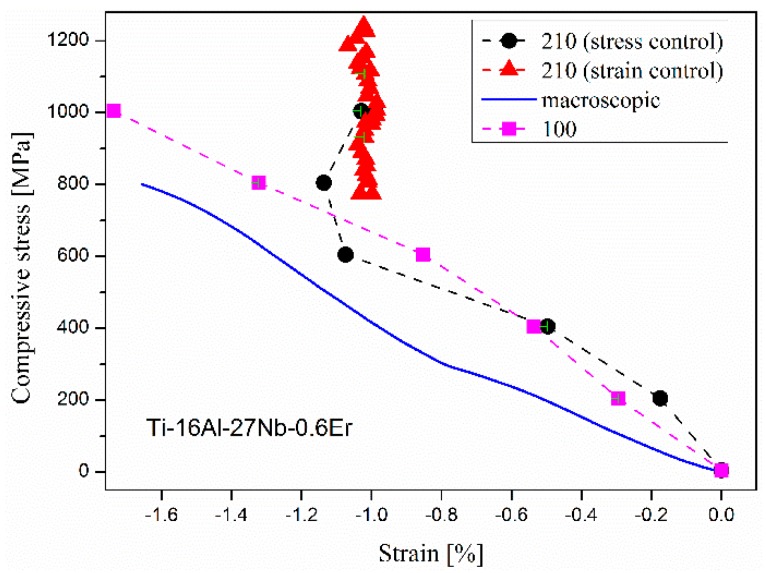
Lattice strain along (210), (100) and compressive macroscopic strain as a function of applied stress. All the error bars are smaller than the symbol size in the figures.

The lattice strain as a function of applied axial stress is shown for both 210 and 100 planes in Figure 4. Elastic anisotropy is clearly observed and elastic moduli of both planes are found to be larger than the average macroscopic modulus. The strong non-linearity of 210 plane at the applied stress of 600 MPa reveals onset of plasticity in this alloy well before the measured macroscopic proof yield stress of 820 MPa. It is also noted that the 210 lattice strain becomes insensitive to the applied stress after loading into the plastic regime, indicating grains with the 210 lattice plane normal parallel to the loading axis could not deform freely in that direction. This plastic anisotropy appears to be related to inter-grain stresses within the sample as the 100 plane shows normal elastic behavior up to 1000 MPa. 

**Figure 5:**
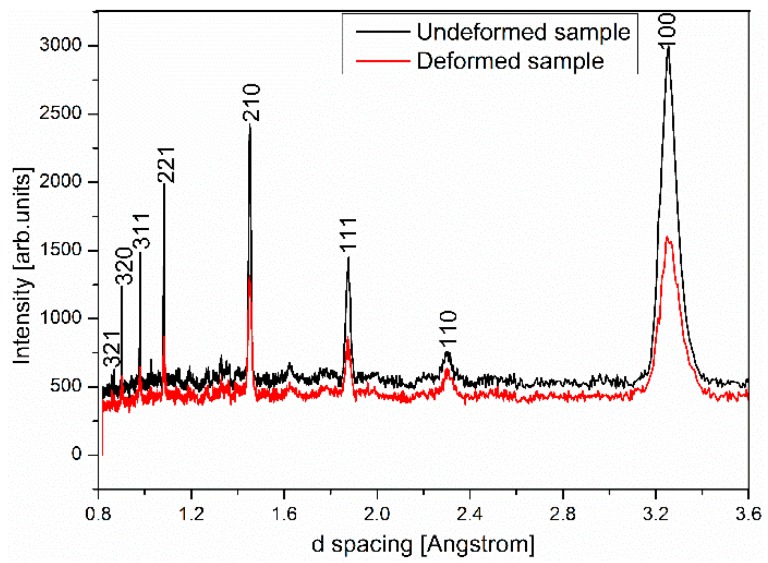
Effects of plastic deformation on NPD patterns (measured with KOWARI diffractometer).

Comparing NPD patterns of the alloy sample before compressive loading and after plastic deformation and unloading, strain induced peak broadening is clearly observed, as shown in Figure 5. Rietveld refinement of the plastically deformed sample was carried out to quantify this effect. Background parameters, diffractometer zero, scale and cell parameter are refined similarly to those in the undeformed sample structure refinement, whereas the atomic parameters and peak profile parameters related to grain size broadening have their values fixed to be identical. Peak profile parameters related to the strain broadening are allowed to refine, which introduces two additional refinement parameters, S_400_ and S_220_ for the cubic B_2_ cell [12,14]. The refined lattice parameter value is identical to that of the undeformed sample within statistical uncertainty. The refined strain broadening parameter values are S_400_ = 19.18 and S_220_ = −0.63, corresponding to a calculated strain of 1.1%. This value is surprisingly close to the measured maximum lattice strain of 1.14% in Figure 4 and indicates that most of this lattice deformation is non-elastic and remains as residual lattice strain after unloading.

On the other hand, the similarity between NPD patterns of undeformed and plastic deformed samples indicates no phase transition happens. The SIMT process proposed in other Ti-Al-Nb alloys [5] was not observed during our in situ stress loading neutron diffraction experiment. 

**Figure 6:**
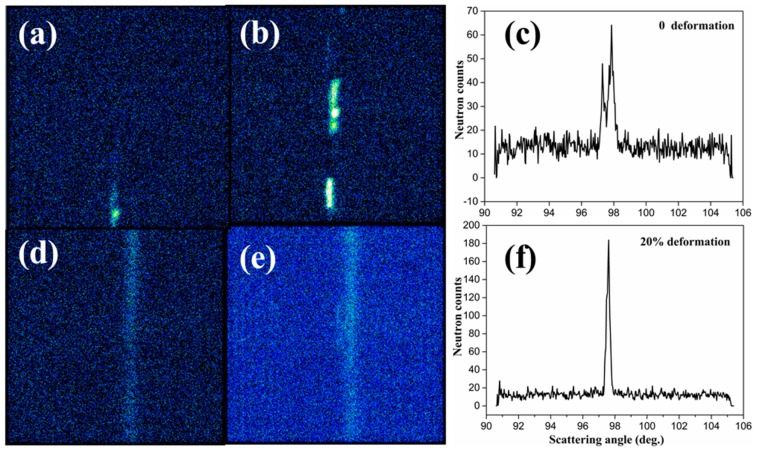
Debye-Scherrer cone of the crystal under approximately (**a**) 0; (**b**) 2%; (**d**) 16% and; (**e**) 20% plastic deformation. The rocking curve measurements for the sample under approximately (**c**) 0 and; (**f**) 20% plastic deformation.

The 2D neutron detector of the KOWARI residual stress diffractometer allows direct view of a section of the Debye-Sherrer cone, as shown in Figure 6. The large intensity variation along the cone, i.e., bright diffraction spots in Figure 6a–e, indicates diffracting grains with {210} normal parallel to the sample rod axis (also the uniaxial loading direction) are quite large in size. A rocking-curve test was performed using the KOWARI diffractometer and the result demonstrates a clear peak splitting in Figure 6c. This suggests that a few large size grains ≥1 mm) dominate the observed diffraction intensities. After the sample experienced 16% and 20% serve plastic deformation, only a single peak was observed (Figure 6f), which indicates the grain refinement during plastic deformation. The grain refinement effect was also shown as the brightness and peak spots’ changes of the diffraction intensity distribution in Figure 6a–e.

**Figure 7:**
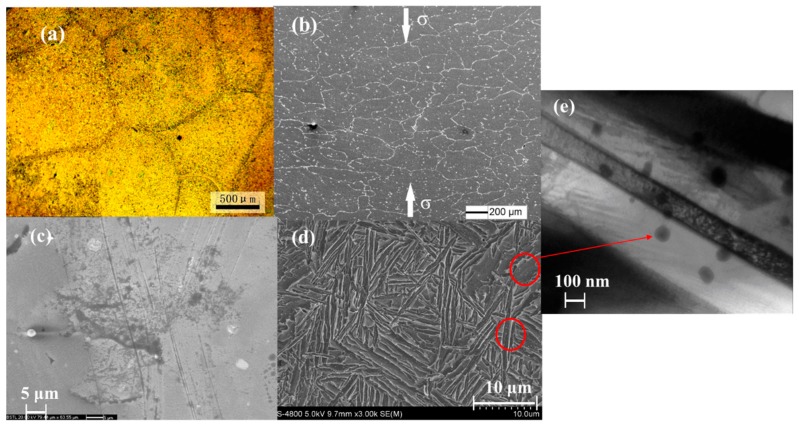
Effects of plastic deformation on microstructure, (**a**) undeformed grains; (**b**) 20% deformed grains; (**c**) multiple slip bands inside the grains; (**d**) microstructure of Ti-16Al-27Nb-0.6Er alloy and; (**e**) (AlTiNb)_2_Er_3_ precipitates.

The SEM observation of the deformed sample verified the large grain size inferred from above. As we know that plastic deformation during compression leads to the decrease of grain sizes which are shown in Figure 7a,b. The deformed grains, which were extent along the orientation perpendicular to the loading direction, are about hundreds of micron meters. From Figure 7c, many slip bands which are parallel or cross to each other after compression can be observed. This was due to the differences of slip system activation, rotation direction and deformation level in different parts within grains during plastic deformation. The microstructure of this alloy can be seen in Figure 7d, composed of B_2_ matrix (black), O plates (gray) and very tiny (AlTiNb)_2_Er_3_ precipitates (white in the circle area, clearly shown in Figure 7e) phases which reveals the Widmanstätten pattern. 

The observed peak splitting as shown in Figure 6b is likely due to slightly different positions of the large diffracting grains within the sampled gauge volume, not evidence of any stress-induced phase transitions. The intensity distribution along the Debye-Sherrer cone does become more uniform into the plastic deformation region as shown in Figure 6d,e, which is attributed to the appearance of much more equiaxed grains under severe plastic deformation [15]. This observed grain refinement effect during plastic deformation is consistent with the previous SEM results. It is believed that the activation of different slip systems and grain refinement might be the structural origin of the novel mechanical properties of this alloy. 

## 4. Conclusions

This study observed the crystal structure of Er doped Ti-16Al-27Nb alloy by using both X-ray and neutron powder diffraction techniques. The in situ neutron diffraction and SEM experiments reveal the lattice strain and microstructure evolution during compressive stress loading. The following conclusions can be drawn:

(1) The primary phase is determined to be the ordered B_2_ structure and its lattice parameters were refined by the Rietveld method;
(2) Due to Bragg reflections satisfying the extinction condition for a bcc cell, h + k + l = odd, and not observed in the measured X-ray diffraction pattern become strong peaks in neutron diffraction patterns;
(3) The decrease of grain sizes during plastic deformation was proposed by both the Debye-Sherrer cone and SEM observations while stress-induced martensitic phase transition was not observed;
(4) It is believed that the activation of different slip systems and grain refinement might be the structural origin of the novel mechanical properties of this alloy.

## Figures and Tables

**Table 1:** Summary of the NPD Rietveld refinement results.

Atom	X	Y	Z	Occupancy
Ti	0	0	0	0.48
Nb	0	0	0	0.52
Ti	1/2	1/2	1/2	0.66
Nb	1/2	1/2	1/2	0.02
Al	1/2	1/2	1/2	0.32
Lattice parameter			a = 3.2508 Å	
Statistical factors wRp = 6.91% χ^2^ = 3.1				
		Rp = 5.25%		
